# Allied Health – 3001. Does treatment of gasteroesophageal reflux disease in patients with allergic rhinitis improve allergic symptoms?

**DOI:** 10.1186/1939-4551-6-S1-P178

**Published:** 2013-04-23

**Authors:** Reza Faridhosseini, Farahzad Jabbari, Afshin Shirkani, Mohammad Reza Zandkarimi, Hadis Yousefzadeh

**Affiliations:** 1 Allergy Research Center, Faculty of Medicine, Mashhad University of Medical Science, Mashhad, Iran; 2 Mashhad University of Medical Science, Iran; 3 Student Research Assembly of Mums, Mashhad University of Medical Science, Mashahd, Iran

## Background

Allergic rhinitis is the most type of allergic disease among population. Its accurate treatment is very important for cutting off allergic Marche. In the other hand, gasteroesophageal reflux disease (GERD) is one of the most gastrointestinal problems among allergic patients like asthma that it might be the treatment conflict. Despite of asthma, we have a few studies about impacts of GERD treatment on symptoms of allergic rhinitis. In this study we evaluated the correlation of GERD treatment and its effects on improvement of allergic rhinitis.

## Methods

This simple randomized single-blind study included 101 patients with moderate to severe allergic rhinitis. Their allergy for aeroallergens was confirmed by skin prick test considered three mm more than negative control. Thirty three of 101 patients (32%) had GERD. For these patients empirically was prescribed 20 mg omeprazol once daily for 6 weeks. Allergic and GERD symptoms evaluated clinically in 5, 10 and 30 days after beginning of treatment.

## Results

Our patients included 48 (46.6%) male and 55 (53.4%) female with mean age 28±11.6 years old. Thirty one of 33 allergic patients by GERD (mean age; 32±11.21 years old) after 6 weeks treatment with omeprazol developed significant improvement for GERD symptoms in 5, 10 and 30 days after beginning of therapy. There was no association between allergic symptoms of these patients and rate of anti-allergic drug consumption (P>0.05).

## Conclusions

This study showed that there was no correlation between empirical treatment of GERD and improvement of allergic symptoms in patients with allergic rhinitis. However further studies with more sample size might be need.

